# A Case of Basal Cell Carcinoma With Metastasis to the Pelvic Bone

**DOI:** 10.7759/cureus.30305

**Published:** 2022-10-14

**Authors:** Gokul Krishnan, Karthik Udupa, Ruchee Khanna

**Affiliations:** 1 Department of Internal Medicine, Kasturba Medical College, Manipal, Manipal, IND; 2 Department of Oncology, Kasturba Medical College, Manipal, Manipal, IND; 3 Department of Pathology, Kasturba Medical College, Manipal, Manipal, IND

**Keywords:** radiotherapy, platinum based chemotherapy, bony metastasis, metastasis, basal cell carcinoma

## Abstract

Our patient initially presented in 2015 with an ulcerative lesion over the scalp. Fine needle aspiration cytology (FNAC) from a regional enlarged lymph node showed features of metastatic poorly differentiated carcinoma and he underwent wide local excision with functional neck dissection. His next visit was after five years in 2020 with pain in the left hip region. Bone marrow biopsy was reported as metastatic carcinoma morphologically consistent with the patient’s known basal cell carcinoma. He received palliative radiotherapy for the same at the hip region followed by platinum-based chemotherapy.

## Introduction

Metastasis of basal cell carcinoma (BCC) is a rare occurrence ranging from 0.0028% to 0.1% [1,2]. Bone is considered the third most common site of metastasis following lymph nodes and lungs [3]. Prognosis is poor for metastatic cases with an average survival of 3.6 years after nodal metastasis with as low as 8-14 months after hematogenous distant metastasis [4]. Treatment for cases of distant metastasis is with a combination of radiotherapy and chemotherapy [5-7]. Our patient initially presented with regional metastases which were treated surgically and later presented with distant metastases to bone. Distant metastases were treated with radiotherapy followed by platinum-based chemotherapy.

## Case presentation

Our patient a 39-year-old male presented in October 2015 with an ulcer over the scalp. It had initially begun as a swelling two years back measuring 2 cm * 2 cm initially. It ruptured while getting his hair trimmed at a salon, ulcerated, and started increasing in size to 10 cm * 15 cm at the time of presentation. Multiple swellings were also noted on the right side of his neck. Fine needle aspiration cytology (FNAC) from a regional enlarged lymph node showed features of metastatic poorly differentiated carcinoma and he underwent wide local excision with functional neck dissection for the same. Following the surgery, our patient received adjuvant radiotherapy with 60 Gy in 30 fractions to affected side neck nodes covering retro auricular, occipital, and level II to IV cervical nodes.

**Figure 1:**
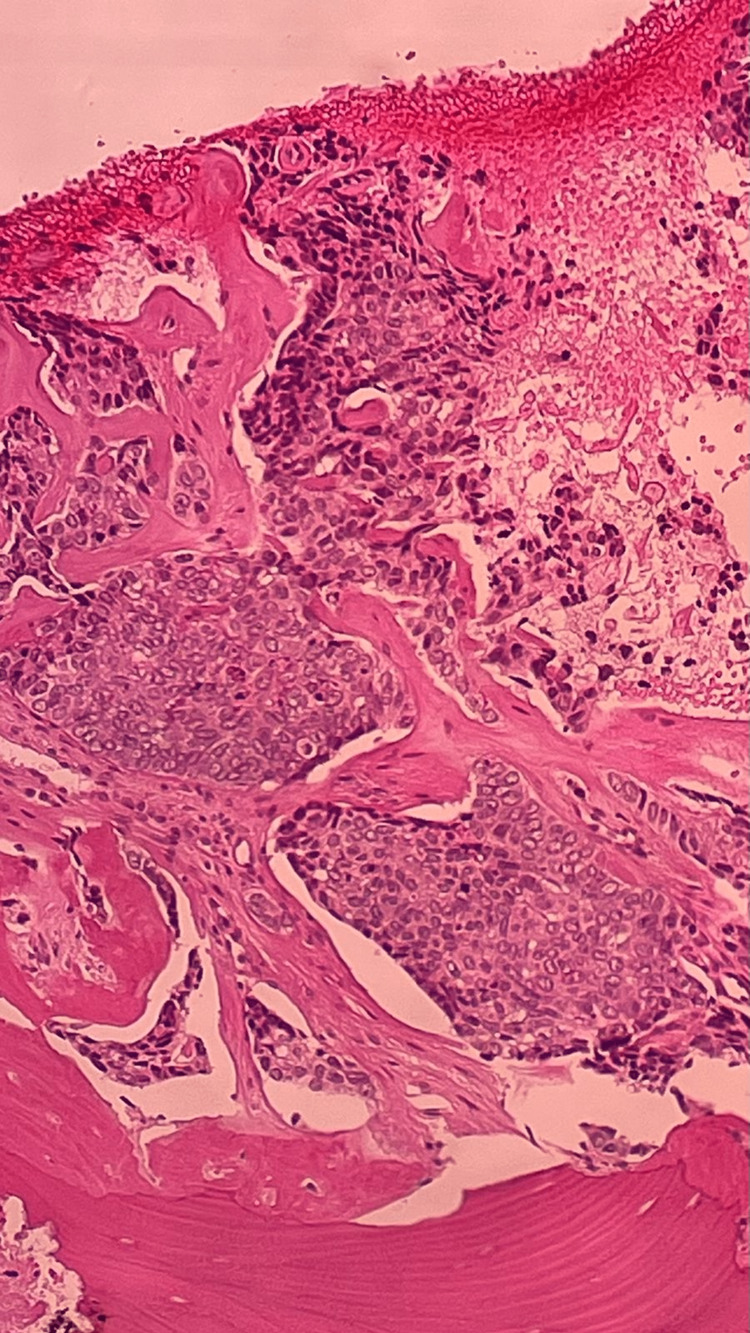
Bone marrow biopsy image Image acquired at 40X magnification Bone marrow biopsy image shows marrow spaces replaced by malignant cells suggestive of basal cell carcinoma

His next presentation was after five years in 2020 with pain in the left hip region. On examination, there was tenderness over the hip. Computed tomography (CT) performed showed sclerotic and lytic areas in bilateral pelvic bones. Bone marrow aspirate of hip bone showed features suggestive of metastatic carcinoma and this was confirmed by bone marrow biopsy which was consistent with basal cell carcinoma (Figure 1).

He was treated with radiotherapy followed by platinum-based chemotherapy. Radiation involved 20 Gy in five fractions and 8 Gy in one fraction. Paclitaxel and carboplatin were the chemotherapeutic agents administered in June 2020.

## Discussion

BCC is a common skin cancer arising from the basal layer of the epidermis and its appendages. These are known to be locally aggressive with low metastatic potential [8]. Metastasis of BCC is rare, ranging from 0.0028% to 0.1% of all cases [1,2]. Criteria to be met to be considered as metastatic BCC as per Lattes and Kessler are (1) the primary tumour must originate from the skin and not mucous membrane, (2) metastasis must be at a site distant from the primary tumour and not a simple extension, and (3) the primary and metastatic lesions must have similar histologic subtypes. These criteria are used to rule out the second primary tumour and locally advanced disease [9].

The spread of disease can be regional or distal. Spread of BCC to regional lymph nodes, adjacent soft tissue including skin and subcutaneous tissue, and muscle in the ipsilateral anatomic region is considered regional metastasis while spread to distant lymph nodes, viscera, bone, brain, or meninges is considered distant metastasis. Our patient had initially presented with regional metastasis to cervical nodes five years back and has presented now with distal metastasis to the pelvic bone. Metastatic BCC spreads by lymphatic and haematogenous routes [4]. For cases with lymph node metastases only, average survival is reported to be 3.6 years after the detection of metastasis [10]. Among cases with haematogenous spread to sites such as bone, liver, and lung, average survival is reported to be 8-14 months [4]. The average time interval between the onset of tumour and metastasis is approximately nine years [2]. In our case, the last contact with our healthcare setting was one month after the diagnosis of metastatic BCC during which he was doing well. Unfortunately, he was lost to follow up from then.

The median age of onset of primary BCC is approximately 45 years. Risk factors for the development of metastasis include a history of early radiation treatment for acne, inadequate radiation treatment of the primary BCC, and large, deeply invasive tumours [11]. The head and neck region is the most common primary site of metastasis [12]. Most common sites of metastasis are regional lymph nodes (60%), lungs (42%), bones (10%) and skin (10%) [3]. We performed metastatic workup in form of CT which showed lytic and sclerotic lesions over bilateral pelvic bones followed by which we decided to go ahead with a bone marrow examination which confirmed the diagnosis. We decided to perform a metastatic workup on our patient as he had a history of malignancy and was complaining of persistent pain over the hip region.

Aggressive BCC of the scalp and ear region may have an increased tendency to metastasize because of the thin skin and increased concentration of large calibre vessels. The depth of invasion is considered more important than the size of invasion for metastasis. This is because larger calibre vessels are present at greater depths. A larger size of tumour embolus is also a prerequisite for metastasis. There are mainly two reasons for the requirement of a large tumour embolus to increase the likelihood of metastasis. Firstly, only a few daughter cells of the embolus might carry the potential. The embolus should consist of a complex of tumour and stromal cells. Secondly, the complex of tumour cells along with stromal cells act as a functional unit enabling successful metastasis [13-16].

According to McCusker et al. in the case of bone metastasis median survival was 12 months as compared to 66 months among those without bone metastasis [4]. However, it was also noted that those presenting with bone metastasis also have metastasis to other sites at the time of presentation. Bone metastasis cases usually present with bony pain, weakness or spinal cord compression features in case of vertebral involvement [17].

Therapy of metastatic basal cell carcinoma depends on the site and extent of the primary tumour and metastasis. For primary tumour and regional metastasis surgical excision is performed. The goal is to obtain clear margins. In the case of distant metastasis, a combination of radiotherapy and chemotherapy is used. Chemotherapy agents used are cisplatin,5-fluorouracil, vincristine, bleomycin, cyclophosphamide, methotrexate, and doxorubicin. According to Pfeiffer et al. [5], cisplatin is the most effective chemotherapeutic agent [5-7]. Hedgehog pathway inhibitor vismodegib is a newer agent found to be effective in advanced basal cell carcinoma [18].

## Conclusions

From this case report, we would like to conclude that bony metastasis though extremely rare is to be included among the differential diagnosis when a person with basal cell carcinoma presents with focal pain or tenderness. Secondly, in such cases of metastatic basal cell carcinoma, platinum-based chemotherapy along with radiotherapy can be considered as a treatment modality.
